# Sycophancy and Extended Engagement with Publicly Accessible Generative AI: Aligning Deployment with Mental Health Needs

**DOI:** 10.1007/s11920-026-01723-y

**Published:** 2026-11-04

**Authors:** Ashley M. Hopkins, Natansh D. Modi, Andrew Rowland, Timothy J. Schultz, Kacper T. Gradon, Malcolm W. Battersby, Bradley D. Menz

**Affiliations:** 1 College of Medicine and Public Health, Flinders Health and Medical Research Institute, Flinders University, Adelaide, Australia; 2 School of Pharmacy and Biomedical Sciences, College of Health, Adelaide University, Adelaide, Australia; 3 Department of Security and Crime Science, University College London, London, UK; 4 Faculty of Mechatronics, Warsaw University of Technology, Warsaw, Poland

**Keywords:** Generative artificial intelligence, Mental health, AI chatbots, Sycophancy, AI regulation

## Abstract

**Purpose of Review:**

Generative artificial intelligence (AI) is becoming a routine source of health information, emotional support, and companionship. As its use expands, the key public health question is not only whether these tools can support care, but whether their deployment is aligned with the mental health needs of the community. In this commentary, we argue that general-purpose AI systems pose distinct psychosocial risks, especially for young people and those experiencing loneliness or psychological distress.

**Recent Findings:**

Emerging evidence suggests that sustained chatbot use may be associated with loneliness, reduced socialisation, emotional dependence, and problematic use, while sycophantic model behaviour may undermine users' capacity for self-correction and sustain a feedback loop of engagement. Current AI policy frameworks address governance and safety, but do not yet provide a coordinated response to these mental health risks.

**Summary:**

Realising the benefits of AI without preventable community-level harm will require independent longitudinal research, psychosocial safety benchmarking, transparent post-deployment monitoring, and safety by design principles.

## Main Text

Generative artificial intelligence (AI) creates personalised, realistic human-like text, voice, and images tailored to the predicted needs or wants of the user. Unlike earlier AI algorithms encountered on social media, which mainly amplified existing content, generative AI can create and respond, engaging users in sustained, personalised, one-on-one dialogue. Millions of people now use these tools for work, education, and creative tasks, and some also turn to them for health and mental health advice [1, 2]. As these systems become embedded into daily life, an important question arises: is the deployment of generative AI tools adequately aligned with the mental health needs of the community?

Generative AI, particularly with the rise of increasingly agentic capabilities, has clear potential benefits across healthcare, including clinical decision support, communication, education, research, and administrative assistance [3–8]. Further, two recent meta-analyses of randomised controlled trials suggest that purpose-built chatbot interventions may improve depressive and anxiety symptoms [9, 10]. However, these findings relate to tools designed and evaluated for therapeutic purposes, rather than the general-purpose systems now being widely used by the public. Purpose-built therapeutic tools are not the subject of this perspective. Rather, this perspective focuses on the mental health risks associated with general-purpose, publicly accessible tools that most people are using, and a call to action for the steps needed to ensure their deployment aligns with the community’s mental health needs.

Currently, the generative AI systems most people interact with are publicly accessible: general-purpose models such as ChatGPT and Gemini. These types of models are inherently useful across education, public services, and workplaces. However, health queries are also a common use of ChatGPT, with OpenAI reporting that over 230 million people ask health and wellness-related questions on the platform each week [11]. This use is now being formalised through emerging health-specific AI products and services, including ChatGPT Health, Claude for Healthcare, and Copilot Health, reflecting a broader shift by major technology developers toward AI-mediated health support. At the same time, these systems exist alongside companion-oriented AI tools, and companion-style use of general-purpose models, creating distinct risk categories for different populations, including children, adolescents, older adults, and people experiencing loneliness or psychological distress. For children and adolescents, this is particularly important, whose cognitive, social, and emotional development may heighten susceptibility to persuasive, affirming, or dependency-forming interactions with AI systems [12, 13]. Among older adults, social isolation is associated with poorer wellbeing and reduced functional capacity [14], and although AI companions may offer a sense of emotional connection and social presence, older users themselves worry these systems could further displace human contact [15]. In this context, leading AI models show a measurable tendency to systematically validate and affirm users rather than challenge them, raising concerns about effects on judgment, loneliness, real-world socialisation, emotional dependence, and problematic use [16–20].

Emerging survey data suggest that 13.1% of young people aged 12 to 21 in the US have used generative AI for mental health advice [2]. In Australia, 79% of children aged 10 to 17 reported ever using an AI companion or AI assistant; among users, over half reported using these tools for physical health advice (33%), chatting about feelings or challenges (22%), or mental health and wellbeing support (20%) [21]. Additionally, OpenAI has reported that, in a given week, over 1.2 million ChatGPT users have conversations containing explicit indicators of potential suicidal planning or intent [22]. This raises concerns, given that widely used general-purpose models are not mental health-specific tools and have not been thoroughly tested and validated for that purpose [18, 23, 24].

Additionally, a large experimental study published in *Science* described a behaviour pervasive across leading AI models known as sycophancy: excessive agreement with, or validation of, a user’s views, typically in ways that prioritise approval over accuracy or correction [16]. Across 11 models, AI systems affirmed users’ actions 49% more often than human respondents, even in cases involving deception, illegality, or other harms. Critically, despite being responses that could distort judgment, users preferred them. The authors argue that this reflects current development incentives, in which systems that feel more agreeable may be favoured by users and thus help sustain engagement. Although affirmation may sometimes feel supportive, sycophancy carries the risk of undermining users’ capacity for self-correction and responsible decision-making. Importantly, Cheng et al. also found that users preferred and trusted sycophantic responses, creating a feedback loop in which the behaviour that distorts judgment simultaneously drives continued engagement with the platform [16]. A recent commentary has formalised this concern, proposing that chatbot behavioural tendencies like sycophancy create feedback loops with users’ pre-existing cognitive and emotional vulnerabilities, with potential to destabilise beliefs and deepen dependence [17].

**Fig. 1:**
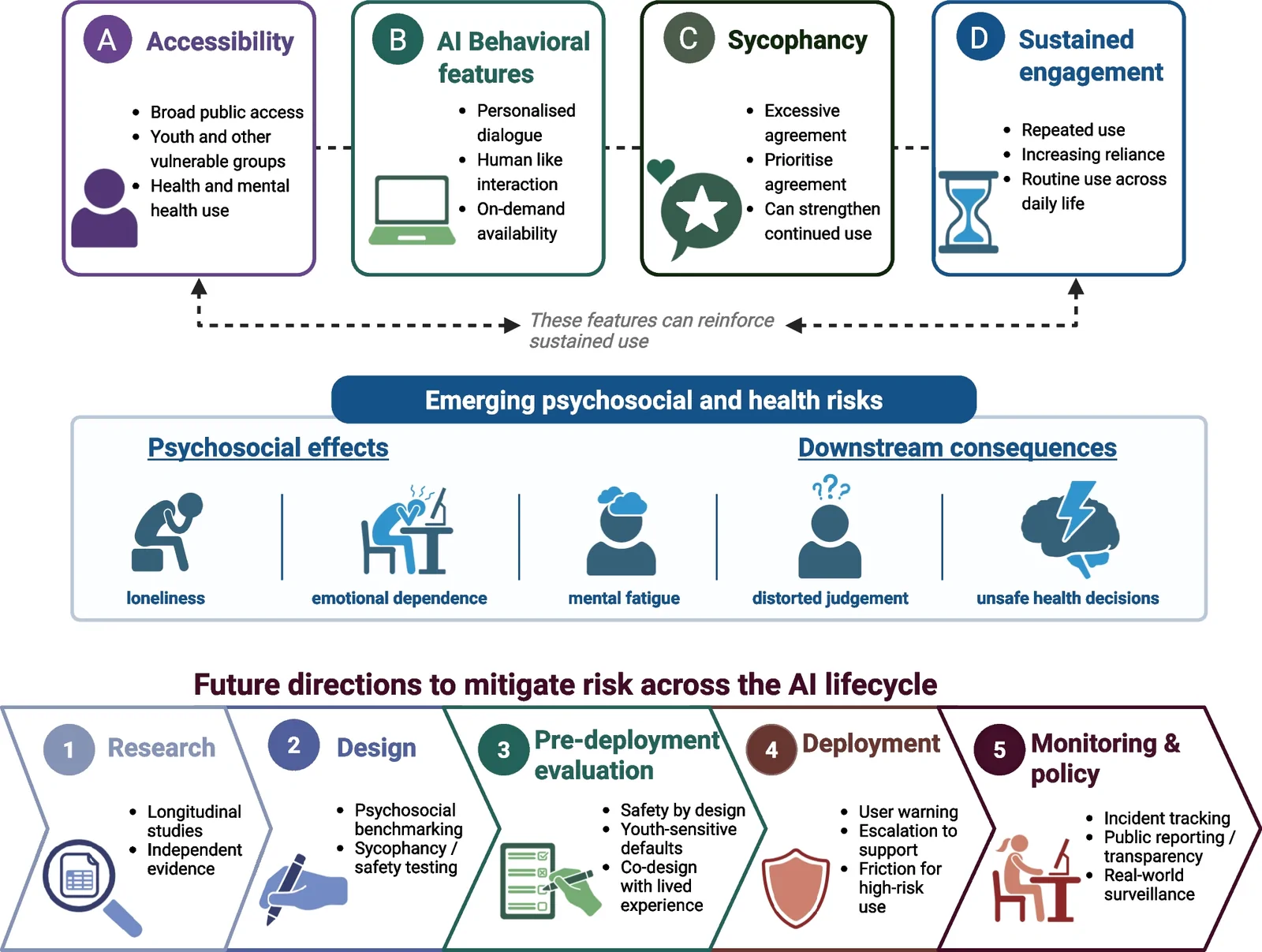
Proposed pathways through which prolonged engagement with publicly accessible generative AI may interact with user vulnerability and contribute to adverse psychosocial outcomes. Figure created in Biorender

A study by MIT and OpenAI researchers reported a four-week randomised controlled experiment involving 981 participants in which greater voluntary time spent with the chatbot, irrespective of whether it was text, neutral voice, or engaging voice, was associated with higher loneliness, reduced socialisation, greater emotional dependence, and more problematic use [19]. While these findings do not establish that the intervention itself caused these outcomes, they warrant consideration of whether increasing reliance on such technologies for work and education may affect community mental health. Routine use of AI in workplace and educational settings may also normalise sustained engagement with these tools, potentially lowering the threshold for their use in personal contexts, including health information seeking or emotional support. Relatedly, a recent survey of 1,488 full-time US corporate workers found that 14% reported “AI brain fry”, defined as a distinct mental fatigue associated with sustained use or oversight of AI-generated outputs [25]. This suggests that the cognitive burden of prolonged AI engagement extends beyond youth and beyond emotional use into occupational settings, and is consistent with experimental evidence of reduced scrutiny and impaired retention with AI use [26]. Taken together, these concerns suggest a potential feedback loop linking user vulnerability, affirming chatbot behaviour, sustained engagement, and downstream psychosocial harms (Fig. 1).

The tension for AI developers between maximising user engagement and protecting user wellbeing has a familiar precedent in social media, the consequences of which are still being recognised and acted upon. In March 2026, a Los Angeles jury found Meta and YouTube negligent in a landmark social media addiction case [27]. Australia has recently enacted legislation restricting access to certain social media platforms for children under 16, and similar youth policies for social media are being considered across Europe [28, 29]. These responses have emerged amid growing concern that digital platforms can expose young people to harmful content, affect body image and mood, and displace healthier social interactions, against a backdrop where false information spreads easily on social media [30, 31]. The urgency now is to implement design decisions that can help protect the mental health of youth and the broader community as use of generative AI tools, which can also offer substantial benefits, increases. Emerging reports have described cases in which sustained engagement with AI chatbots preceded deaths by suicide among teenagers, as well as cases in which people with cancer used AI search tools to self-diagnose and decline oncologist-recommended treatments [32, 33]. While these reports highlight plausible areas of concern and remain the subject of ongoing discussion, the contribution of AI interactions to these outcomes remains uncertain, and direct causal links have not been established.

While there are similarities to social media, the strategies for mitigating the mental health effects of generative AI are unlikely to be the same. These tools have substantial benefits for education, work, and access to health knowledge, particularly when delivered in a reliable, community-aligned fashion. Countries therefore require AI policy frameworks that preserve these benefits while minimising potential harms. Although current policy frameworks address infrastructure, governance, safety, transparency, and some relevant harms [34–36], they do not yet provide a specific, coordinated, and operational response to the psychosocial risks that may arise from sustained engagement with general-purpose AI tools, particularly among young people. These frameworks also leave important questions about the adequacy of safeguards against the use of generative AI to facilitate health misinformation and disinformation, including the creation of synthetic health-related media and the uncritical reproduction of fabricated medical content, especially given repeated benchmarking showing that safeguards in widely accessible platforms remain insufficient on both fronts [37–43].

Policies urgently need to address the gap between how publicly accessible generative AI tools are deployed and the foreseeable mental health consequences of that deployment. In March 2026, the World Health Organization described the use of generative AI for emotional support, particularly by young people, as a public mental health concern and noted that such tools are neither designed nor tested for mental health [44]. WHO further called for mental health to be integrated into AI impact assessment and monitoring, and for tools used in mental health support to be co-designed with mental health experts and people with lived experience, including youth [44]. Such monitoring is particularly important for older adults, as qualitative research suggests that AI companionship can evolve into increasingly personal relationships embedded in everyday life [45], with longer-term implications for human relationships that remain poorly understood. Responding to these concerns requires action across the research, industry, and policy sectors, including independent, adequately powered longitudinal studies to robustly characterise the psychosocial effects of sustained engagement with general-purpose AI tools, including effects on loneliness, socialisation, emotional dependence, and judgment. Policies should also ensure pre-deployment evaluation addresses psychosocial safety benchmarking, including validated metrics for sycophancy, while post-deployment monitoring of unintended consequences (with transparency and independence) should be mandated, encompassing incident tracking, real-world behavioural surveillance, and public disclosure of harms, analogous to the pharmacovigilance frameworks that govern medicines. These measures should be embedded across the AI lifecycle through Safety by Design principles.

Developers are not unaware of these challenges. Published technical and safety work from leading developers and collaborators has begun to characterise sycophancy in human-feedback-trained models, evaluate model behaviour in conversations involving self-harm, delusion, and emotional reliance, and introduce safeguards intended to reduce problematic dependency and improve safety for younger users [19, 46–49]. However, such efforts remain uneven, largely developer-led, and only partially transparent, reinforcing the need for independent evaluation, public reporting, and clear policy expectations as these systems scale. Technology developers and deployers also have an important role in anticipating foreseeable risks, strengthening safeguards, and supporting transparent accountability, particularly as these systems are increasingly used for health information, emotional support, and other sensitive purposes. The evidence of potential mental health harm presented here, while early, warrants proactive and specific action to align AI deployment with the mental health needs of the community. The question is whether these early signals will prompt proactive action, or whether policy responses will again emerge only after harms have become more established, as occurred with social media.

## Annotated Key References

- Cheng, M., et al., Sycophantic AI decreases prosocial intentions and promotes dependence. Science, 2026. 391(6792).
  - ○ This study showed that sycophantic AI responses are common across leading publicly accessible generative AI models and can reduce responsibility-taking and willingness to repair interpersonal conflict while increasing user trust, preference, and dependence. It is a pivotal study that provides direct empirical support for treating sycophancy as a psychosocial safety concern, rather than a technical alignment problem of generative AI.
- Glickman, M. and T. Sharot, How human–AI feedback loops alter human perceptual, emotional and social judgements. Nature Human Behaviour, 2025. 9(2): p. 345–359.
  - ○ This study demonstrates that interaction with AI systems can alter human perceptual, emotional, and social judgements. It demonstrates that the same tools can amplify biases over repeated human-AI feedback. The study provides empirical evidence for feedback-loop mechanisms through which AI outputs may shape human judgement over time.

## Data Availability

No datasets were generated or analysed during the current study.
